# Unlocking the potentials of digital twins for optimal healthcare delivery in Africa

**DOI:** 10.1093/oodh/oqae039

**Published:** 2024-09-16

**Authors:** Ibraheem Olasunkanmi Qoseem, Musa Ahmed, Hamzat Abdulraheem, Muhammad Olaitan Hamzah, Mohamed Mustaf Ahmed, Bonaventure Michael Ukoaka, Olalekan John Okesanya, Jerico Bautista Ogaya, Olaniyi Abideen Adigun, Aniekan Michael Ekpenyong, Don Eliseo Lucero-Prisno III

**Affiliations:** Department of Medical Laboratory Science, Kwara State University, P.M.B 1530 Ilorin, 23431, Malete, Kwara State, Nigeria; Department of Medical Laboratory Science, Kwara State University, P.M.B 1530 Ilorin, 23431, Malete, Kwara State, Nigeria; Department of Medical Laboratory Science, Kwara State University, P.M.B 1530 Ilorin, 23431, Malete, Kwara State, Nigeria; Department of Medical Laboratory Science, Kwara State University, P.M.B 1530 Ilorin, 23431, Malete, Kwara State, Nigeria; Faculty of Medicine and Health Sciences, SIMAD University, Hamarjadid District, Warshadaha Street, PO Box 630, Mogadishu, Somalia; Department of Internal Medicine, Asokoro District Hospital, No 31, Julius Nyerere Crescent, Asokoro, Aso 900103, Abuja, Nigeria; Department of Public Health and Maritime Transport, University of Thessaly, Volos, 382 21, Greece; Department of Medical Technology, Institute of Health Sciences and Nursing, Far Eastern University, Nicanor Reyes Street, Sampaloc, Manila, 1008, Metro Manila, Philippines; Center for University Research, University of Makati, JP Rizal Ext, West Rembo, Makati City, 1215, Metro Manila, Philippines; Department of Medical Laboratory Science, Nigerian Defence Academy, Kaduna, PMB 2109, Nigeria; Research Unit, Global Health Focus Africa, 19, Area F, Pyakasa, 900107, Abuja, Nigeria; Department of Global Health and Development, London School of Hygiene and Tropical Medicine, Keppel Street, London, WC1E 7HT, United Kingdom; Research and Development Office, Biliran Province State University, Leyte, P. Inocentes St, Naval, 6543, Biliran, Philippines; Research and Innovation Office, Southern Leyte State University, Concepcion St, Sogod, 6606, Southern Leyte, Philippines

**Keywords:** digital twins (DT), healthcare applications, predictive health tracking, personalized medicine, digital health, health equity

## Abstract

Advances in big data analysis, the Internet of Things and simulation technology have led to a surge in interest in digital twin technology, which creates virtual clones of physical entities across several industries. The technological revolution with digital twins, incorporating Internet of Things, big data analysis and simulation technologies, holds the potential for predictive insights, real-time monitoring and increased operational efficiency across the healthcare industry. This paper explores the potential of digital twins to improve healthcare delivery and health outcomes in Africa. It examines their applications in various health sectors, explores their feasibility and highlights the potential challenges associated with their implementation while proposing sustainable recommendations.

## INTRODUCTION

Digital technologies permeate and transform service delivery across all sectors. However, only a few nations, particularly the global south, leverage the cutting-edge potential of technological innovations. This is partly due to limited access to quality Internet services, which plagues nearly half of the world’s population [1]. A digital divide has been observed between developed and developing countries [2]. Exclusions and disparities in income, sex and age are additional factors that exacerbate pre-existing inequalities, particularly for less fortunate populations in the digital sphere [1]. Research across Africa has revealed significant health inequalities. These disparities, multifaceted in etiology, affect diverse populations unequally, with worse health outcomes for people in rural areas, racial and ethnic minorities and women [3, 4]. The rapid advancement of digital health innovations has significantly reduced these gaps. For quality healthcare service delivery, telehealth, smart wearable technologies, Internet of Medical Things, artificial intelligence (AI), machine learning, blockchain and remote patient monitoring have emerged to bridge the gap and change the tide [5, 6]. For instance, telehealth has been studied to effectively close population disparity gaps, facilitating the delivery of healthcare services to people of color and racial differences, gender, key populations and the underserved [7]. A digital twin (DT) is a virtual model that accurately reflects a physical object, system or process [8]. The model is continuously updated with data from its real-world counterpart using sensors, Internet of Things (IoT) devices or other data collection methods. DT allows for real-time monitoring, simulation, analysis and optimization of the physical entity it represents. Health systems provide improved patient care, predictive analytics, clinical operation optimization and opportunities for training and simulation by utilizing real-time data integration, sophisticated analytics and virtual simulations [8]. DT is beneficial for customized and personalized medicine. By digitally simulating the human body, medical practitioners can implement more successful interventions and enable the prevention, early identification and focused treatment of various disorders. DT can assist in product design, optimization and testing [9]. For patients and healthcare delivery, DTs enable healthcare providers to gather and analyze patient data from various sources, including electronic health records (EHRs) [10]. With the impact observed across spheres such as engineering, aeronautics and manufacturing, there is potential for DTs in the healthcare sector. This paper aims to illuminate the potential of DTs to foster healthcare delivery and enhance health outcomes. It analyzes its applications across various health sectors, explores their applicability and highlights the potential challenges associated with its use in the African healthcare sector. It also suggests recommendations that may support the uptake of technology on the continent.

### DT technology in healthcare: an overview

The advantages of digital health twins have been widely explored and recognized. It has emerged as a powerful tool for improving healthcare delivery in Africa. From biomedical engineering to clinical medicine, its potential in big data analysis, communication and simulation technology has raised interest among experts in its application and research [10, 11]. DTs are digital replicas of physical entities, either living or nonliving, with the ability to anticipate future events and dynamically adjust to variations in real-time data. Through closed-loop contact with their surroundings, these intelligent and developing technologies can continuously predict future states and optimize processes [12]. They possess the potential to manage patients as independent entities across diverse healthcare scenarios. The technologies essential for DTs can be categorized into data-driven statistical models and multiscale knowledge-integrated mechanical models. The numerical model computes the structural performance, whereas the analytical model facilitates the structural analysis. An AI model trained with samples and numerical data derives real-time structural insights from sensor data. DT technology has radically changed several industries, including healthcare, where it has the potential to significantly improve diagnoses [13].

### Role and relevance of DT and digital health technologies

Healthcare systems are being revolutionized by the growing use of diverse health information technologies, which have opened new channels for administration and communication [14]. For instance, ‘Industry 4.0’, also known as the fourth industrial revolution and ‘intelligent industry’, signifies a new era of interconnectedness, automation, machine learning and real-time data, which emerged in 2011 [15]. It also encompasses cyber-physical systems, the IoT, cloud computing and cognitive computing. As an advancing frontier in industrialization, nearly all sectors, including healthcare, are leveraging the impact of this technology. DT technology is an enabling concept for implementation in Industry 4.0. There are several prospects for digital technology to transform the health care industry. For instance, IoT solutions may supply real-time data, and strong digital infrastructure can manage and safeguard massive data flows. Furthermore, command centers, AI and machine learning enhance flow and decision-making assistance [16]. As individual technological units, these innovations can provide solutions to challenges in the health industry. However, when integrated into a single model, advanced solutions can be achieved with a short processing time and turn-around time. The development of DTs aims to make this feasible [17].

### DTs’ applications in healthcare

DTs are crucial for reducing errors in healthcare procedures and minimizing adverse outcomes in patient management [18]. A notable application of DT is Safety 4.0, an evolution in the approach to workplace safety driven by the technologies and principles of Industry 4.0. It leverages advanced technologies, such as IoT, big data, AI and augmented reality, to enhance safety measures, making workplaces safer and more efficient. DTs can be integrated into the Safety 4.0 framework to accurately manage complex safety processes and reduce human error [19]. This synergism becomes difficult in clinical patient management, especially in times of frequent medical errors and mismanagement. During the COVID-19 pandemic, DTs have become essential tools for technology-driven projects and virtual interventions. They play a significant role in vaccination program development, vaccine supply chain optimization, acceptance rate prediction and immunization hurdle identification [20]. Healthcare organizations often face challenges, such as privacy concerns, data exchange limitations and an overwhelming amount of patient data. The DT technology has transformed clinical research and medicine by enabling users to ask intelligent questions, gain better answers and extract actionable insights from data while safeguarding real individuals’ privacy and health [21]. This technology enables robust information and knowledge management, leading to improved decision-making, optimized research outputs and enhanced patient care.

Technologies that improve sleep, physical activity, nutrition and mental health have been developed to achieve health and well-being goals [22]. DTs have proven useful in managing healthcare costs while promoting behavioral changes through modern digital health interventions [23]. For instance, the Coach, a user-centered smart coach that guides and instructs users based on individual assessments of their skills, helps clients make necessary adjustments to their performance and posture [24]. ClouDTH is a cloud-based healthcare system that uses data from wearable medical devices to create a convergence of virtual and real spaces, allowing elderly individuals to manage their health more effectively [23]. The field of geriatric medicine has also received wide application of DT as it has been used to facilitate customized treatments, remote health data monitoring and active patient participation in their care [25]. DTs enable early data gathering and anomaly detection, enhancing response times and interventions [21]. They optimized hospital efficiency by simulating changes in workflow and managing critical care resources, as shown during the COVID-19 pandemic. [26]. This comprehensive application of DTs in healthcare exemplifies their transformative impact on patient care and healthcare administration [27].

The fields of drug discovery and biomanufacturing have also witnessed the application of DTs. Efficiency has been instilled into the pharmaceutical production process by reducing costs, timelines, and attrition rates associated with traditional methods [28]. Integrating DT modeling with computer-aided drug discovery and machine learning techniques has scaled up the screening of ADME-Tox properties, thereby accelerating the identification and validation of successful drug targets. This technology has led to the introduction of drugs for diseases, such as HIV and cancer [29]. Partnerships between Atos, Siemens, and pharmaceutical companies such as GSK and Takeda Pharmaceuticals demonstrate their impact on optimizing drug manufacturing processes. In addition, DTs enhance biomanufacturing by simulating biochemical reactions and optimizing process parameters using predictive models [30]. Industry 4.0, which has further simplified drug manufacturing by integrating AI, robotics and IoT, aims to efficiently produce innovative, customized pharmaceutical products [31].

Utilizing precise, patient-specific anatomical simulations, DT technology has improved surgical planning by reducing errors and improving the results. For example, in cardiac surgery, surgeons can perform intricate cardiac procedures such as transcatheter aortic valve replacement (TAVR) using DT models such as HeartNavigator [12, 32]. Additionally, prosthetic choices and implantation procedures can be investigated through ‘virtual TAVR’ simulations to facilitate informed decision-making before surgery. In orthopedic surgery, surgeons have leveraged DTs to research and evaluate medical implants before fixation [33, 34]. These models aid in the selection of the best postoperative care plans and stabilization techniques, based on the unique needs of each patient. DTs of trabecular bone are created using cutting-edge methods, such as Deep Convolutional Generative Adversarial Networks. They help simulate vertebroplasty operations and forecast their effects on vertebral fractures [35]. Furthermore, in oncology, DTs are used to forecast the risk of vertebral fractures in patients with metastatic spinal cancer after stereotactic body radiotherapy. The precision and efficacy of surgical operations across a range of medical professions are greatly improved by DT technology, which makes comprehensive presurgical planning and risk assessment possible [27].

### Challenges associated with the use of DTs in Africa

The novelty of DT in Africa presents an initial resistance to acceptance and integration compounded by peculiarities in the continent’s health systems. The nascent stage of DT technology, lack of clear understanding and competency in technical and practical aspects and limited case studies and business models hinder its progress [36]. Integrating multiple evolving technologies, such as 3D simulations, IoT, Industrial IoT, AI, big data, machine learning and cloud computing, is essential for DT. However, these technologies are still being developed and alien to the continent. The substantial investment required for DT infrastructure can be prohibitively expensive, particularly in Africa, where healthcare funding is often limited [37].

The availability, quality and security of data are also a problem, as many African healthcare systems lack robust data-management frameworks. Additionally, there is a lack of standardized rules and regulations governing DT use in healthcare, leading to ambiguity and uncertainty in its application. The complexity and scale of DT projects require advanced tools and infrastructure that are not readily available in many African settings [38]. Another significant challenge is the complexity in selecting the most suitable platform for specific needs. The variety of software solutions available, such as Predix by General Electric, Azure Digital Twin by Microsoft and IBM Watson, can overwhelm stakeholders, complicate decision-making and potentially lead to suboptimal choices [39]. This is in a context where most specialists in the use of these technologies are based in the global north, further exacerbating the ability to utilize these platforms on the continent.

Time and cost are significant barriers to the widespread adoption of DT in Africa’s healthcare sector. The development of ultra-high-fidelity computer models and simulation processes is time-consuming and labor-intensive, and requires significant computational power. The high cost is further exacerbated by the need to integrate existing systems with sensors for data collection and establish a high-performance IT infrastructure [40]. Analysts like Marc Halpern highlight the resource-intensive nature of DT concepts and the extreme scale of investment required, estimating trillions of dollars and extensive timeframes for comprehensive implementation. This sheer amount of investment is challenging in an often-cash-strapped healthcare sector, thereby necessitating thorough cost–benefit analyses before undertaking DT projects [41].

Accurate real-time updates depend on high-fidelity two-way synchronization, which can be challenging for large-scale systems owing to the need for extensive resources and reliable IoT connections. Ensuring interoperability with current software used in various healthcare processes, such as inventory and patient management systems, is another significant challenge [38]. Creating and managing DT software calls for dedicated staff, and ongoing investment is required to stay updated with the developments in big data, machine learning and IoT [42]. Standardization is essential for uniformity, security and efficient data access, especially in critical industries such as healthcare. However, the lack of established laws and regulations for evolving DT-related technologies such as big data and AI has led to fragmented approaches and hindered cohesive adoption [43]. Data-related issues such as privacy, confidentiality and data ownership are also significant challenges. Without clear data-sharing policies, data silos can emerge, leading to inconsistencies and synchronization issues [44]. Data interoperability becomes complicated when multiple DTs at different hierarchical levels generate and rely on varied data types, resulting in significant challenges in data integration and consistency. It can be challenging to combine and synchronize data from several sources into a coherent DT strategy, because healthcare systems frequently contain a variety of data sources and formats. Cybersecurity risks are further compounded by the extensive data required for DTs, making them attractive cyber-attack targets [36]. Life-cycle mismatching is another critical challenge for DT technology, especially for products and infrastructure with long life cycles. The validity and compatibility of the software used to create and maintain their DTs often exceed the longevity of these physical assets, raising the risk of software obsolescence and dependency on specific vendors for updates and new versions [45]. Strategic planning and investment are needed to develop sustainable and flexible DT solutions that can evolve alongside their physical counterparts in Africa [41].

### Recommendations

Addressing the challenges of DT implementation in the African healthcare landscape requires a myriad of approaches. There needs to be strengthening of the digital health infrastructure on the continent. This requires investing in the improvement of Internet connectivity across both urban and rural areas, so that healthcare facilities have access to the Internet. This may warrant partnerships between the government and private sector to enhance broadband access. The availability of high-quality Internet access would further strengthen the digitization of health records, thereby facilitating data management and integration. As most countries in Africa lack quality EHR systems, adopting a policy focus on DTs may serve as an impetus for the government to substantially fund projects to digitize health records, transforming them from paper-based to digital forms [46]. This is key to achieving digital twinning, especially in the areas of precision and individualized medicine programs.

Africa has a critical shortage of human health resources. With the sector struggling to attract individuals to provide healthcare services, the DT policy agenda could be a way to address this challenge. Furthermore, DT scalability in Africa can only be achieved when there is a critical mass of individuals with experts in this area. Thus, governments, in partnership with the private sector and universities, would need to invest in existing or establishing new training programs aimed at building capacity in digital health, data analytics and digital twinning. This provides the opportunity to attract individuals from the technology sector to address the digital gap in healthcare [6], thereby aligning with the need for cross-sectoral collaboration to achieve various global goals, such as Universal Health Coverage, health-related Sustainable Development Goals and Agenda 2063 of the African Union. Leveraging existing technology is crucial for actualizing the DT policy agenda in Africa. Simple technologies such as mobile health and telemedicine have been utilized to improve access to quality healthcare, especially in rural areas and hard-to-reach communities [47, 48]. These tools can effectively serve as a bridge between patients and healthcare providers, while also functioning as a means for collecting and transmitting healthcare data. This enables real-time data gathering, owing to the widespread use of mobile phones on the continent. This ensures that the data collected are not only restricted to urban areas where there is a higher likelihood of EHRs, but also to underserved areas, ensuring health equity is achieved. A multidisciplinary strategy involving the partnership of data scientists, technologists, legislators, patients and patient advocates is needed to create strong frameworks, standards and guidance for creating, applying and implementing DTs in the African healthcare landscape. To protect the rights of people with DTs, maintain data security and privacy and promote fairness and transparency in the use of data in society, effective governance frameworks must be in place [49]. For DTs to be widely used in healthcare, ethical standards, legal framework compliance and maintaining accountability and openness are essential. Thus, existing data protection regulations will need to be reviewed to acknowledge the role of technologies such as DT in advancing quality patient care and, most importantly, to guarantee the safety of patients and the users of this technology.

## CONCLUSION

In conclusion, the DT technology is reshaping industries such as the healthcare sector using big data analysis, IoT and simulation technology, providing real-time monitoring, predictive insights and operational efficiency improvements. DTs enable the dynamic simulation of treatment plans, predictive health trajectory tracking and personalized medicine interventions. Despite challenges, such as data interoperability and privacy concerns, DTs have demonstrated their value in driving innovation, cost-effectiveness and sustainability. They can address the peculiar challenges that the healthcare sector in Africa have been grappling with. However, there are significant issues that need to be addressed for actualizing the benefits of DTs in Africa. Governments and policymakers are required to take the lead in guaranteeing substantial funding opportunities in collaboration with the private sector and global partners to ensure that the necessary infrastructure, policies and governance frameworks are established to reap the benefits of DTs on the continent.

## Data Availability

Now new data were generated for this study.
